# Editorial: Computational predictions, dynamic tracking, and evolutionary analysis of antibiotic resistance through the mining of microbial genomes and metagenomic data, volume II

**DOI:** 10.3389/fmicb.2023.1203297

**Published:** 2023-05-10

**Authors:** Qi Zhao, Jian Li, Alfred Tay, Liang Wang

**Affiliations:** ^1^School of Computer Science and Software Engineering, University of Science and Technology Liaoning, Anshan, China; ^2^School of Public Health and Tropical Medicine, Tulane University, New Orleans, LA, United States; ^3^The Marshall Centre for Infectious Diseases, Research and Training, University of Western Australia, Perth, WA, Australia; ^4^Laboratory Medicine, Guangdong Provincial People's Hospital (Guangdong Academy of Medical Sciences), Southern Medical University, Guangzhou, China; ^5^Centre for Precision Health, School of Medical and Health Sciences, Edith Cowan University, Perth, WA, Australia

**Keywords:** antibiotic resistance, microbial genomes, metagenomic data, computational prediction, evolutionary analysis

Antibiotic resistance has emerged as a critical global health challenge, posing a significant threat to human and animal health. The rapid development of antibiotic resistance among pathogens has reduced the efficacy of existing treatments, leading to an urgent need for novel therapeutic strategies. One promising avenue of research involves mining microbial genomes and metagenomic data to uncover novel antimicrobial agents and a better understanding of the resistance mechanism. Advances in sequencing technologies and computational approaches have enabled the rapid characterization of these genomes, revealing a vast array of novel genes and gene clusters with potential antimicrobial properties. Moreover, metagenomic data provides a comprehensive picture of the complex microbial communities present in various environments, such as soil, water, and the human gut. These datasets contain millions of previously unknown genes, many of which may hold the key to uncover new antimicrobial agents. By conducting large-scale metagenomic studies, scientists can delve into the untapped reservoir of genetic diversity and uncover novel antimicrobial compounds with unique modes of action.

By comparing the genomes of antibiotic-resistant and susceptible strains, researchers can identify the genetic elements responsible for conferring resistance, such as mutations or gene acquisition events. Under this Research Topic, we sought to highlight an exciting set of groundbreaking work by frontline investigators, which mainly focused on implementing computational methodologies to get an in-depth understanding of microbial antibiotic resistance. We are pleased to note that our Research Topic has attracted contributions from several highly regarded researchers in this field around the world, including from China, USA, United Kingdom, and Germany. From the received submission five were accepted for publication after rigorous review.

In this special issue, Pei et al. collected 2,035 clinical *Klebsiella pneumoniae* (KP) isolates from a tertiary hospital in China. A genome-wide association study was conducted via a Bayesian-based method, including single nucleotide polymorphisms (SNPs) and gene sequence screening. A total of 28 and 37 potential genes were identified to have an association with imipenem and meropenem resistance, respectively. These results indicate complex mechanisms of carbapenems resistance and further investigation of Carbapenem-resistant KP (CRKP)-related factors are warranted to better understand their contributions to carbapenems resistance.

Although the mechanisms of Influenza A Virus (IAV) infection have been unraveled, the underlying complex mechanisms evolved by gut microbiota in order to induce host immune response following IAV infection remain ambiguous. Bhar et al. compared the performance of a novel maximal-clique-based community detection algorithm for weighted undirected networks (MCCD-WN) with other existing algorithms using three sets of benchmark networks. Their network analysis reveals a faster recovery of the infected cohort after the second IAV infection and provides insights into the crucial roles of *Desulfovibrionaceae* and *Lactobacillaceae* families in combating IAV infection.

To understand the colonization and community functional dynamics of the microbiota, based on responses to host immune processes during the normal and dysbiotic establishment of the gut, Peters et al. investigated 91 fecal samples collected over the first 90 days of life of 17 hospitalized premature infants. This study provides a unique approach to examine the temporal expansion and resilience of microbial colonization, as it allows simultaneous examination of both host and microbial metabolic activities. Monitoring the growth of bacterial cultures is one of the most common techniques in microbiology.

Worth et al. developed a completely novel way of obtaining bacterial growth curves based on the classification of scanned images of cultures rather than using spectrophotometric measurements. They tested the accuracy of this tool, ScanGrow, and demonstrated one of its applicability options by analyzing the effect of several concentrations of different antibiotics on bacterial growth curves.

Finally, Dai et al. explored the toxin-antitoxin (TA) systems by mining the publicly available genomic sequences from the NCBI SRA database. Their work confirmed a wealth of TA genes in the unexplored *Pseudomonas aeruginosa* pan-genomes, expanded the knowledge on *Pseudomonas aeruginosa*, and provided methodological tips on large-scale data mining for future studies.

As a summary, we want to thank all the authors who contributed their original work to our special issue and the reviewers for their valuable comments. We would like to express our sincere gratitude to the editorial office of Frontier in Microbiology, for their excellent support and for providing us with this opportunity to hold this hot topic issue successfully.

## Author contributions

QZ drafted the manuscript. LW revised the draft. AT and JL made substantial contributions to the work through in-depth discussion. All authors proposed the Research Topic theme, made a direct and intellectual contribution to the work, and approved the final version for publication.

